# Enhancing the Sensitivity of Nanoplasmonic Thin Films for Ethanol Vapor Detection

**DOI:** 10.3390/ma13040870

**Published:** 2020-02-14

**Authors:** Marco S. Rodrigues, Joel Borges, Filipe Vaz

**Affiliations:** Centro de Física, Universidade do Minho, Campus de Gualtar, 4710-057 Braga, Portugal; mprodrigues@fisica.uminho.pt (M.S.R.); fvaz@fisica.uminho.pt (F.V.)

**Keywords:** thin films, gold nanoparticles, titanium dioxide, sputtering, plasma etching, plasmonic sensing, optical transmittance change, optical signal quality

## Abstract

Nanoplasmonic thin films, composed of noble metal nanoparticles (gold) embedded in an oxide matrix, have been a subject of considerable interest for Localized Surface Plasmon Resonance (LSPR) sensing. Ethanol is one of the promising materials for fuel cells, and there is an urgent need of a new generation of safe optical sensors for its detection. In this work, we propose the development of sensitive plasmonic platforms to detect molecular analytes (ethanol) through changes of the LSPR band. The thin films were deposited by sputtering followed by a heat treatment to promote the growth of the gold nanoparticles. To enhance the sensitivity of the thin films and the signal-to-noise ratio (SNR) of the transmittance–LSPR sensing system, physical plasma etching was used, resulting in a six-fold increase of the exposed gold nanoparticle area. The transmittance signal at the LSPR peak position increased nine-fold after plasma treatment, and the quality of the signal increased six times (SNR up to 16.5). The optimized thin films seem to be promising candidates to be used for ethanol vapor detection. This conclusion is based not only on the current sensitivity response but also on its enhancement resulting from the optimization routines of thin films’ architectures, which are still under investigation.

## 1. Introduction

Thin films incorporating gold (Au) or silver (Ag) nanoparticles and exhibiting plasmonic behavior have received significant attention over the past years due to their high sensitivity to different dielectric environments [1,2]. Several microstructures can be achieved when different nanoparticles are embedded in different host dielectric matrixes, making it possible to tailor the Localized Surface Plasmon Resonance (LSPR) extinction band [3,4,5,6,7,8,9,10]. Thus, the range of possible applications for these nanomaterials is wide, from decorative colored coatings [11] to the detection of single molecules [12,13]. These molecules may be detected as shifts of the LSPR band [14] when they are adsorbed near the nanoparticles, since they produce a slight change in the refractive index. Due to the short decay length (exponential decay of the evanescent field with the increasing distance from the surface of the plasmonic metal nanoparticle) of localized surface plasmons [15,16,17,18,19,20], the sensing zone of these LSPR sensors is highly localized near the nanoparticle, limiting the size of the sensing volume to around 30 nm from the surface of the nanoparticle [21,22]. 

A titanium dioxide (TiO_2_) matrix and noble metal nanoparticles (Au or/and Ag) may be used independently in sensing applications [23,24,25,26,27,28,29,30], but it is known that their sensing capabilities are enhanced when they are combined [31]. Moreover, by using a mixture of these materials, the mechanical stability of TiO_2_ is combined with the plasmonic sensing properties of Au. These plasmonic thin films, consisting of noble metal nanoparticles embedded in an oxide matrix, have proven to be mechanically strong materials [32] for applications that require contact with external elements, such as decorative coatings and sensors. While for decorative applications these type of thin films are usually prepared with a thickness above 400 nm, the thickness of LSPR thin film sensors should be below 100 nm [33], so that most nanoparticles embedded in the film can be influenced by the presence of analyte molecules. To enhance the sensing capabilities, a physical [5,10] or chemical [34] etching process can be employed, thus making the nanoparticles semi-exposed, but still anchored in the oxide matrix.

Ethanol is an important volatile organic compound that has been recently investigated for the production of fuel cells [35,36,37], as a substitute of hydrogen and methanol, which are more toxic and dangerous to transport. Moreover, the production of ethanol from biomass is an efficient process [38]. It is thus important to have a new type of optical ethanol vapor sensor that can be stable at high temperature and that can be used inside the channels that feed an engine working with an ethanol fuel cell. Most used sensors rely on electrical measurements [39,40,41,42], so the huge advantage of an optical sensor is that there is no need for electrical wiring inside the conduit, thus reducing the risk of ignition and explosion. A visible light beam can simply enter the conduit through an optically transparent window, interact with the plasmonic thin film, then leave through another transparent window, and finally interact with a detector.

In this work, composite Au–TiO_2_ thin films were prepared in a three-step, fully physical and straightforward process with no by-products, involving: i) reactive DC magnetron sputtering with a Ti–Au target, ii) in-air annealing, and iii) Ar plasma etching. The composition, morphology, and optical response of the thin films were analyzed. Finally, the effect of a surface plasma treatment on the sensitivity of the films against ethanol vapor was studied.

## 2. Materials and Methods 

**Figure 1:**
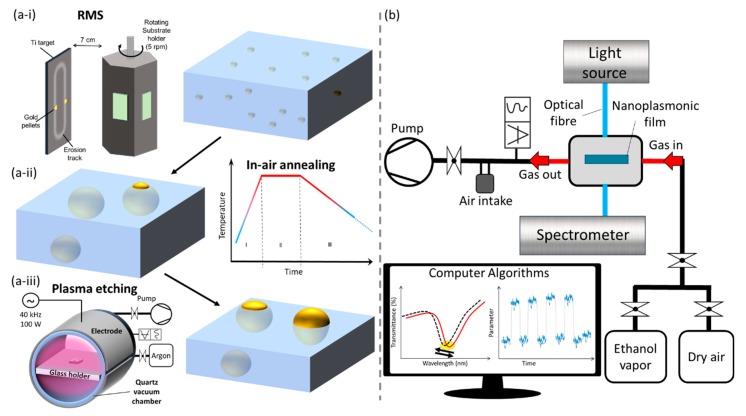
(**a**) Simplified production methods for the plasmonic thin-film sensors in three steps, (**a-i**) Reactive Magnetron Sputtering (RMS) showing the target and the sample holder geometry inside the vacuum chamber), (**a-ii**) in-air annealing process in three phases (I—heating ramp of 5 °C /min, II—plateau at 400 °C for 5 h, and III—free cooling), and (**a-iii**) argon plasma etching process in a Diener Zepto vacuum chamber. (**b**) Simplified schematics of the optical Transmittance–Localized Surface Plasmon Resonance (T-LSPR) sensing system.

Plasmonic thin films were produced by reactive DC magnetron sputtering using a pure titanium target (200 × 100 × 6 mm^3^, 99.99% purity), with gold pellets (two half disks with 4.5 mm diameter, 99.9% purity) incrusted in the erosion track (detailed view in Figure 1a–i), in a custom-made 60 L vacuum chamber [33]. All depositions were performed after a vacuum of approximately 4 × 10^−4^ Pa was reached inside the reactor. During the sputtering process, the atmosphere was composed of a mixture of Ar (flow of 25 sccm, partial pressure of 3.8 × 10^−1^ Pa) and O_2_ (flow of 4.5 sccm, partial pressure of 5 × 10^−2^ Pa). The plasma was ignited, using a current density of 100 A.m^−2^ for 18 min. The grounded sample holder was used in rotation mode (5 rpm), at 7 cm from the target. No external heating was used. Glass substrates were used for optical and morphological characterization and for ethanol vapor sensitivity tests. Before all depositions, the surface of the substrates was cleaned by applying a plasma treatment for 15 min using a Low-Pressure Plasma System (Zepto Model with a 40 kHz RF generator, Diener Electronic, Ebhausen, Germany), with a power of 100 W in an 80 Pa argon atmosphere. 

In a second step, to induce the necessary morphological changes of the Au nanoparticles, needed to tailor the thin films sensitivity, an annealing treatment was performed at 400 °C for 5 h in air (Figure 1a–ii). Then, finally, to partially expose the nanoparticles, while keeping them anchored to TiO_2_, the films were etched using an argon plasma treatment system (Figure 1a-iii) for 1 h, a process optimized in a previous work [10].

The morphological features of the films before and after the plasma treatment were observed by Scanning Electron Microscopy (SEM) using an AURIGA Compact FIB–SEM from ZEISS (Oberkochen, Germany), operated at 5 kV. To make the samples conductive, a 0.5 nm layer of Pt was deposited. The Au nanoparticles, exposed after plasma etching treatment, size distributions (Feret diameter, aspect ratio, and the nearest neighbor), were investigated using SEM micrographs and analyzed in MATLAB (version R2018a, The MathWorks, Inc., Netick, MA, USA).

The composition was characterized by Rutherford backscattering spectrometry (RBS). RBS measurements were made in a chamber with three detectors installed: a standard detector at 140° and two pin-diode detectors located symmetrically to each other, both at 165°. Spectra were collected for 2 MeV 4He+. The angles of incidence were 0° (normal incidence) and 25°. The RBS data were analyzed with the IBA DataFurnace NDF v10.0a (CTN, Instituto Superior Técnico, Lisboa, Portugal) [43], double scattering [44], and pileup [45].

The ethanol vapor sensitivity test was conducted in a custom-made optical system (Figure 1b) that allows transmittance measurements in different atmospheres (T–LSPR sensing system). It consists of a vacuum chamber, a rotary vane pump, several gas inlets, a tungsten light source, and a spectrometer (HR4000, Ocean Optics, Edinburgh, UK). Cycles of a dry-air atmosphere and pure-ethanol atmosphere were then used inside the chamber containing the plasmonic thin film, while measuring the transmittance spectra (Before introducing the test gases, a primary vacuum of 2 × 10^2^ Pa was achieved, and each gas was introduced until a pressure of 2.5 × 10^4^ Pa was reached. The spectrometer acquisition parameters were set to 4 ms integration time and an averaging of 500 scans, giving a spectrum every 2 seconds, and for each atmosphere a total number of 60 transmittance spectra were acquired. The transmittance spectra were then analyzed using an algorithm written in MATLAB that calculates the exact position of the LSPR peak (both wavelength and transmittance, by fitting a polynomial function to each spectrum) and the transmittance at several discrete wavelengths. Then, the shifts of these quantities between both atmospheres were estimated, and the signal-to-noise ratio (SNR—ratio between the average shift and the standard deviation) were calculated. Optical Transmittance Change (OTC) was also determined considering the difference between the average transmittance spectra in dry air and the average transmittance spectra in ethanol.

## 3. Results and Discussion

### 3.1. Plasmonic Thin Films Characterization

The chemical analysis of the as-deposited nanocomposite thin films was performed by RBS, and the Au atomic concentration was found to be of 6 at.%, dispersed in a nearly stoichiometric TiO_2_ matrix (30 at.% of titanium and 64 at.% of oxygen). The thickness was estimated at 100 nm. To make the films even more economically sustainable compared to previous works [10,33], both the thickness and the Au concentration were lowered to a threshold where they maintained a well-defined LSPR extinction band.

**Figure 2:**
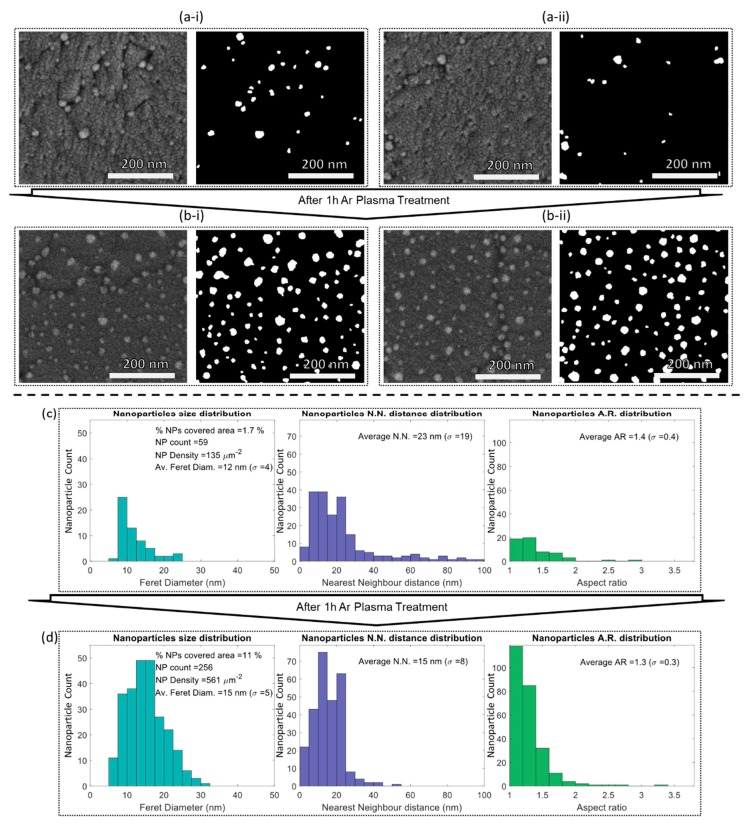
SEM top-view micrographs in two different areas of the film’s surface, before (**a-i**,**a-ii**) and after (**b-i**,**a-ii**) the “uncovering” of the Au nanoparticles by plasma treatment. Statistical analysis of the exposed nanoparticles (size, nearest neighbor, and aspect ratio distributions) before (**c**) and after (**d**) plasma treatment.

To study the effect of plasma treatment on the morphology of the thin film’s surface, top-view SEM were acquired, before (Figure 2a-i) and after plasma treatment (Figure 2b-i). As expected, the striking feature that can be extracted from these micrographs is that the visible Au nanoparticles at the film’s surface had increased. 

After examining the images in MATLAB, nanoparticles size, nearest neighbor, and aspect ratio were depicted in the histograms of Figure 2a-ii and b-ii. After plasma treatment, the nanoparticle count increased four times, and the Au-covered area increased six times, thus reaching a density of 561 nanoparticles/µm^2^. The average size (Feret diameter) of the nanoparticles increased slightly from 12 to 15 nm, and their distribution became slightly broader. The partial uncovering of the nanoparticles brought new neighbors to the surface, and so the nearest-neighbor distribution became narrower after plasma treatment. As a consequence, the nearest neighbor became much closer, and its average distance changed from 23 to 15 nm. The nanoparticles aspect ratio (AR) distribution became slightly narrower, and the nanoparticles became slightly rounder (AR of 1.3). Thus, after plasma treatment, the resulting surface of the thin films presented rounder nanoparticles with a diameter of 15 nm, separated from each other by 15 nm. 

**Figure 3:**
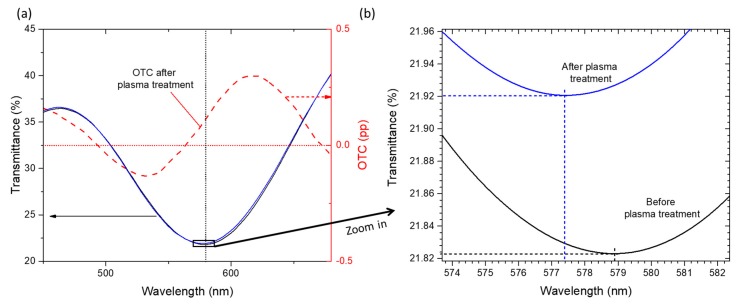
(**a**) Transmittance of the films before and after plasma treatment, with the resulting Optical Transmittance Change (OTC); (**b**) Zoom of the spectra showing a blueshift of the LSPR peak of 1.5 nm.

The optical response of the films was also examined before and after Ar plasma treatment (Figure 3). As can be seen from Figure 3a, the transmittance spectra changed slightly, presenting a 1.5 nm blueshift in the wavelength position of the LSPR peak and a shift around 0.10 pp in the transmittance coordinate (Figure 3b). From the OTC (calculated by subtracting the spectra before and after plasma treatment) of Figure 3a, one can conclude that the higher positive change between both spectra occurred around 619 nm, where the transmittance was increased by 0.30 pp, and the higher negative change was around 532 nm, with a transmittance decrease of 0.13 pp. The blueshift in the LSPR band was also indicated by the positive slope between 532 and 619 nm. This blueshift resulted from an overall response of the thin film due to the exposure of Au nanoparticles, which became in contact with air. Since air has a lower refractive index than the TiO_2_ matrix (where the nanoparticles were still partially anchored), the effective refractive index of the surrounding media was reduced, which explains the shift of the LSPR band [10].

### 3.2. Ethanol Vapor Sensitivity

**Figure 4:**
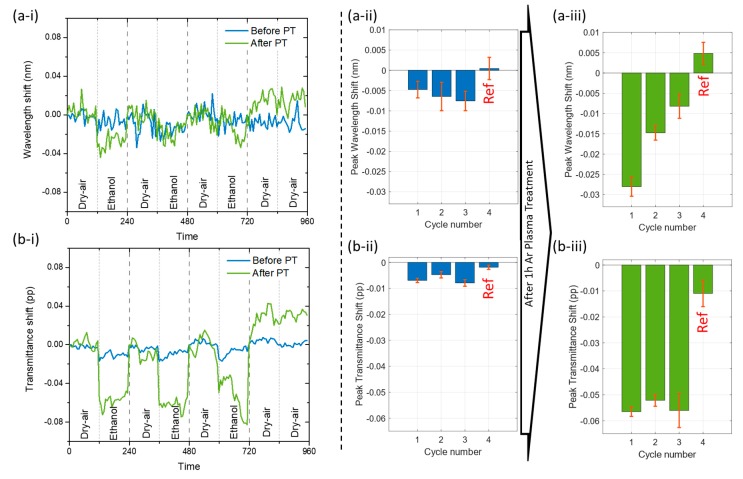
LSPR peak position monitoring of dry-air and ethanol vapor cycles, before (blue) and after (green) 1h Ar plasma treatment show (**a**) the wavelength and (**b**) the transmittance coordinates shift of the LSPR peak. (**a-i,b-i**) Over-time measurements. Before plasma treatment, an average wavelength blueshift of only (**a-ii**) 0.006 nm gave rise to (**a-iii**) a three-fold increase corresponding to 0.017 nm, while the transmittance shift was increased from (**b-ii**) 0.007 pp to (**b-iii**) 0.055 pp (nine-fold increase).

The room-temperature ethanol vapor sensitivity of the films, before and after plasma treatment, was tested against dry air, by measuring their transmittance spectra in each atmosphere for several cycles, with 60 spectra taken in each cycle (Figure 4), as explained in the experimental section.

LSPR peak shifts were measured for both wavelength (Figure 4a) and transmittance (Figure 4b) coordinates. The cycles between each gas were perfectly visible over time (Figure 4a-i,b-i), and it is obvious that, after plasma treatment, the response of the plasmonic thin films was enhanced, especially in the transmittance coordinate. The LSPR peak wavelength coordinate absolute shift was enhanced from 0.006 nm (Figure 4a-ii) to 0.017 nm (Figure 4a-iii), corresponding to a three-fold increase, and the transmittance absolute shift was also enhanced from 0.007 pp (Figure 4b-ii) to 0.055 pp (Figure 4b-iii), thus resulting in a nine-fold increase. The reference cycle (last cycle in the graphs, marked as Ref in red), where the measurement was made with two atmospheres of dry air only, was always significantly different from the test cycles (ethanol and dry air), and no change was measured, thus confirming the different response of the plasmonic thin films to ethanol and dry-air atmospheres. From these results, one can conclude that only an adsorption process occurred [46,47], since the plasmonic thin films recovered in each cycle. As demonstrated in this work and in other related works [5,10], the argon plasma treatment caused a small etching to the film’s surface, exposing the nanoparticles. Several cycles were done, and the ethanol vapor sensitivity was maintained, but other measurements are required to confirm long-term stability.

**Figure 5:**
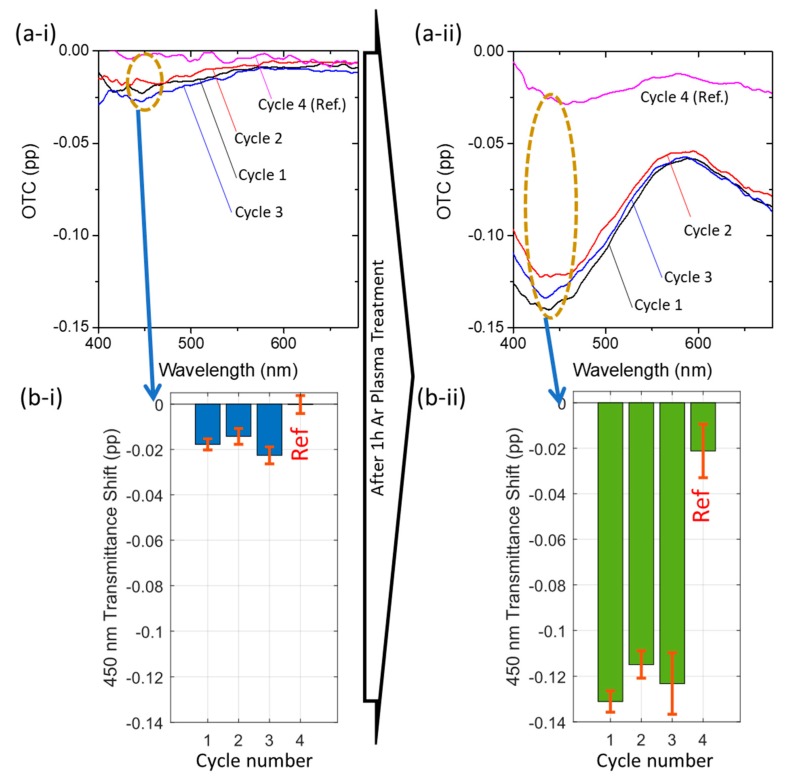
(**a**) Optical transmittance change of the full spectra and (**b**) transmittance shift at 450 nm, between dry-air and ethanol atmospheres, before (**a-i**,**b-i**) and after (**a-ii**,**b-ii**) plasma treatment. The transmittance shift at 450 nm was estimated to increase from 0.018 pp to 0.123 pp, a seven-fold increase.

For a detailed analysis of the full visible spectra, when the films were exposed to dry air and ethanol, the OTC was calculated before (Figure 5a-i) and after (Figure 5a-ii) plasma treatment. For both thin films, the higher transmittance change (decrease in transmittance) occurred near 450 nm, and not at the LSPR peak position (~578 nm), so the transmittance shift was analyzed at 450 nm (Figure 5b-i,b-ii). Similarly, a seven-fold signal increase was achieved with plasma treatment, from 0.018 pp to 0.123 pp.

**Figure 6:**
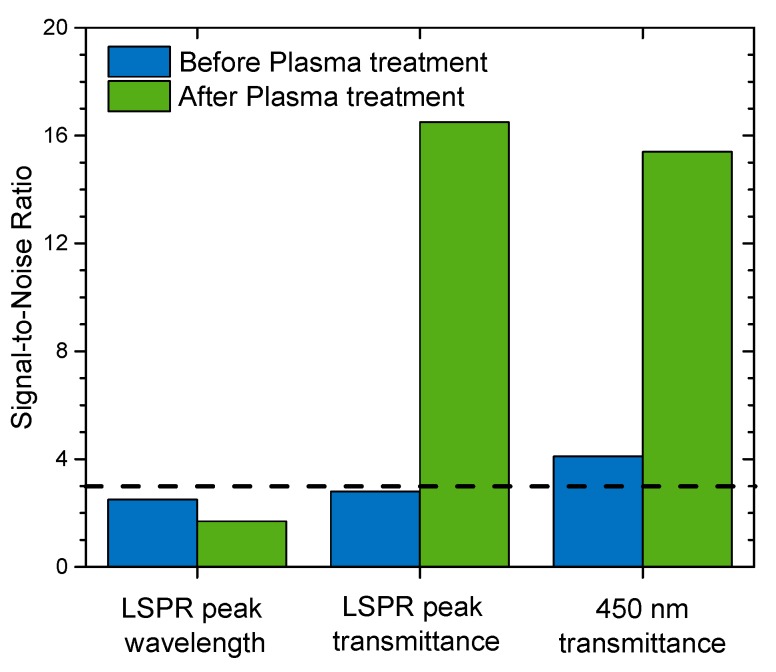
Signal-to-noise ratio (SNR) of the several measured signals, before and after Ar plasma treatment. The dashed line sets the threshold of the minimum SNR needed for detection.

To evaluate the signal quality, the SNR was calculated for each case (see Figure 6). Regarding the LSPR peak wavelength coordinate shift, the SNR was always below the detection limit (SNR < 3) [48]. Therefore, although the LSPR peak wavelength signal was enhanced, the quality of the signal remained practically unchanged after plasma treatment. Nonetheless, considering the transmittance coordinates at the LSPR peak, the SNR was significantly improved from 2.8 to 16.5 (a six-fold increase), making this parameter more suitable for a good-quality measurement. For the transmittance shift at 450 nm, the SNR was also improved, increasing from 4.1 (before plasma treatment) to 15.4 (after plasma treatment), a little bit less than the transmittance shift in the LSPR peak position. Thus, although the signal at 450 nm was higher when compared to the transmittance at the LSPR peak position (0.123 pp at 450 nm and 0.055 pp at the LSPR peak position), the latter was more stable, thus giving a better SNR. 

## 4. Conclusions

In this work, a plasmonic thin film composed of Au nanoparticles dispersed in a TiO_2_ matrix was optimized to be sensitive to ethanol vapor. An innovative preparation method was used, consisting of three main steps, namely, i) reactive magnetron sputtering, ii) in-air thermal treatment, and iii) low-pressure Ar plasma etching. The last step allowed the nanoparticles to become partially exposed at the film’s surface, favoring the interaction with the analyte molecules (ethanol), thus resulting in improved film’s sensitivity. This sensitivity enhancement resulted in a signal that was increased by a factor of 9 in the LSPR transmittance coordinate and a factor of 7 in the transmittance measured at 450 nm. There seems to be a correlation between the SNR and the exposed Au area, since they both had a six-fold enhancement. The SNR results confirmed that the transmittance measurements at the LSPR peak (SNR = 16.5) and at 450 nm (SNR = 15.4) can be used as detection signals of the sensor.
